# Iron deficiency‐induced thrombocytosis presenting with blue toe syndrome

**DOI:** 10.1002/ccr3.8596

**Published:** 2024-04-03

**Authors:** Khaled Aboujabal, Khaled Sadek, Koutaibah Obaid, Bisher Sawaf, Elmukhtar Habas, Mhd Baraa Habib

**Affiliations:** ^1^ Department of Internal Medicine Hamad Medical Corporation Doha Qatar; ^2^ Department of Cardiology Hamad Medical Corporation Doha Qatar

**Keywords:** blue toe syndrome, iron deficiency, microvascular thrombosis, thrombocytosis

## Abstract

Although the etiology of blue toe syndrome is varied, the association between blue toe syndrome and iron deficiency‐induced thrombocytosis (IDIT) has not been well established. We report the case of a 38‐year‐old Saudi male who presented with blue toe syndrome and laboratory investigations revealed severe thrombocytosis secondary to iron deficiency. The patient was managed with analgesics, antiplatelets, anticoagulation, intravenous fluids, and iron supplementation. Subsequently, his symptoms resolved within a few days. IDIT is crucial to consider as a possible cause of microvascular thrombosis disorders, especially in patients with severe thrombocytosis.

## 1 INTRODUCTION

Iron deficiency anemia is a prevalent condition with an estimated global prevalence of 1.24 billion people. It is estimated that iron deficiency without anemia accounts for at least double the number mentioned.^1^ Iron deficiency‐induced thrombocytosis (IDIT) causing an overproduction of platelets, is a known complication of iron deficiency.^1^ Thrombocytosis, defined as a platelet count exceeding 450,000 per microliter, can be categorized as spurious, reactive, or clonal depending on the underlying etiology and pathogenesis.^2^ Iron deficiency anemia is a prominent cause of reactive thrombocytosis, linked to a 4%–6% risk of thrombotic complications, particularly venous in nature.^2^ The link between iron deficiency and thrombocytosis has been reported in various studies which found that iron deficiency anemia was associated with a higher prevalence of thrombocytosis.

Blue toe syndrome (BTS) is a rare manifestation of acute peripheral arterial occlusion characterized by sudden onset of painful, bluish discoloration of one or more toes, usually secondary to microvascular obstruction.^3^ Several etiologies have been proposed for BTS, including thromboembolic, vasoconstrictive diseases, inflammatory processes among others.^3^ However, the association between BTS and IDIT is not well established.

In this case, we presented a patient with an initial presentation of BTS and was subsequently diagnosed with severe thrombocytosis secondary to iron deficiency. The aim of this report is to highlight the importance of considering IDIT as a potential cause of thrombotic events particularly BTS.

## 2 CASE PRESENTATION

### 2.1 Case history and physical examination

A 38‐year‐old man with a history of Laparoscopic Sleeve Gastrectomy 10 years prior to admission presented to the emergency department complaining of severe pain and discoloration of his left foot toes. He reported that he was in his usual state of health until 7 days prior to admission, when he suddenly developed left‐sided upper and lower limb motor weakness that lasted for around 10 min, with no associated residual weakness. Afterward, on the same day, he developed left foot pain. The pain rated nine out of ten, burning in nature, radiated to the left thigh, partially relieved by paracetamol. He developed blue‐gray discoloration of the first, second, and third left foot toe 3 days prior to presentation. Subsequently, the first and second toe discoloration and pain improved but the third toe remained obviously blue and painful.

The patient denied any other past medical history or any personal history of similar previous episodes. He also did not report any history of Stroke, TIA, thrombosis, or coagulopathies. Moreover, other than Leukemia in his brother, he denied any family history of Coagulopathies, Vasoconstrictive disorders, autoimmune disorders, or hematological disorders. As for his social history, he works as a businessman, reported significant work‐related stress, a current 17‐pack‐year smoking history, but he denied any alcohol use or drug use.

**FIGURE 1:**
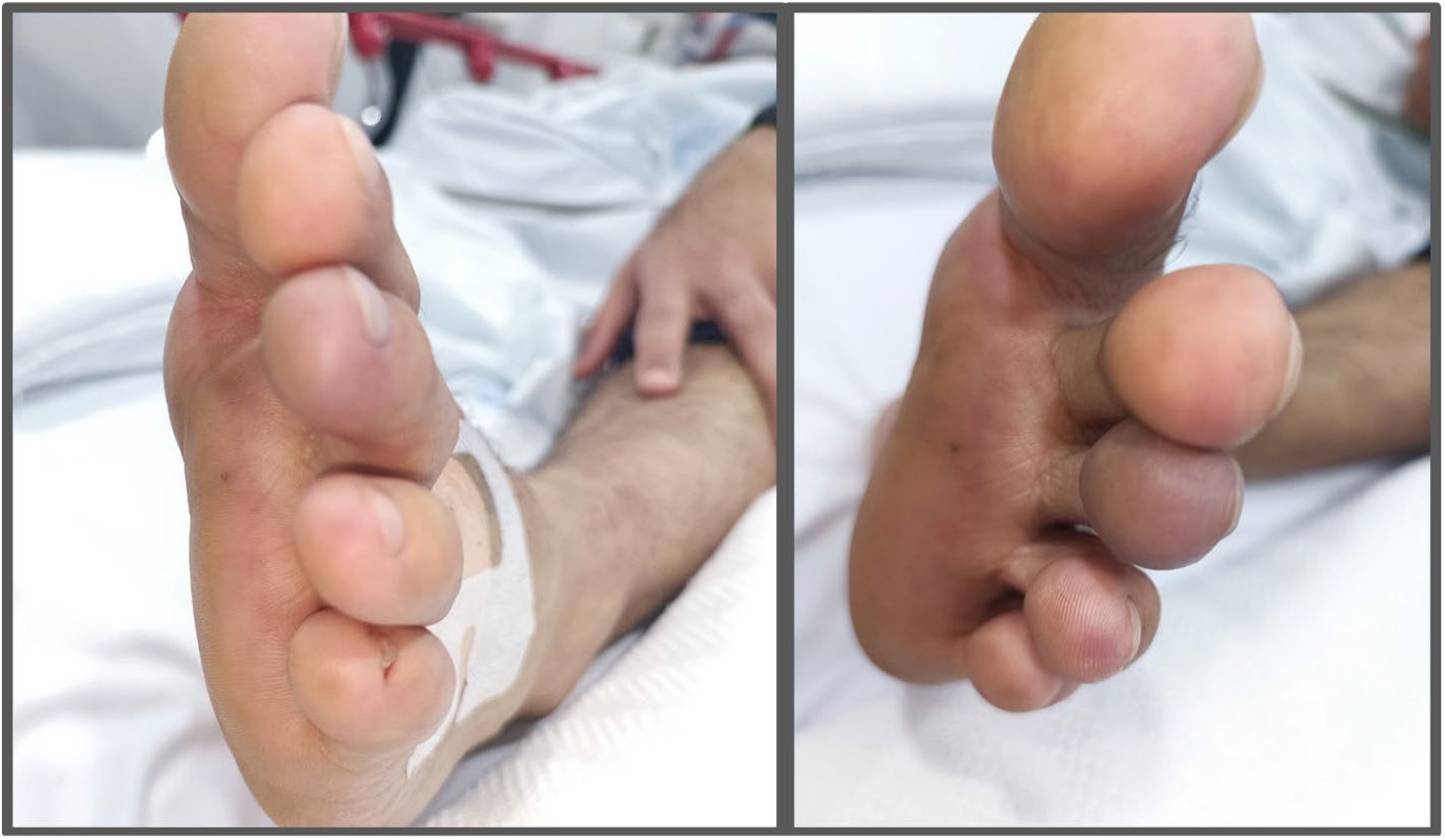
Left third toe of the patient showing bluish discoloration with severe tenderness to palpation on physical examination.

On presentation, he was afebrile, with a heart rate of 89 bpm and his blood pressure was 109/60 mmHg, with no meaningful difference between either arm. However, blood pressure was not measured in the lower limbs. Clinical examination revealed severe tenderness of the first and second toes with bluish‐purple discoloration of the third toe (Figure 1). Peripheral pulses were palpable in both feet, and the contralateral limb did not have any evidence of ischemia. The cardiovascular examination was unremarkable, with audible S1 and S2, with no added sounds suggestive of valvopathy audible. Moreover, Carotid Auscultation did not reveal any murmurs. Abdominal examination revealed a soft and lax abdomen, with no tenderness, overlying skin changes, organomegaly, or renal artery bruits. No skin lesion was identified on examination, and mucous membranes were intact with no discoloration or lesions. The musculoskeletal exam of the major joints (neck, shoulder, elbow, hip, knee, and ankle) did not reveal any tenderness or limitation in the range of motion in the joints. Per‐rectal exam revealed normal color stool and was negative for melena or fresh bleeding per rectum.

### 2.2 Investigations and treatments

Laboratory tests (Table 1) revealed severe microcytic hypochromic anemia, (hemoglobin of 5.1 g/dL and mean corpuscular volume of 57.2) and thrombocytosis, with a platelet count of 1144,000 per microliter. Anemia work‐up revealed severe Iron Deficiency Anemia (Ferritin of 2.3 μg/L, Iron of 2 μmol/L, Iron saturation of 3%, and total iron binding capacity of 75 micromole/L). Tests for thrombophilia, which included Protein C deficiency, Protein S deficiency, Antithrombin III deficiency, and serum homocysteine levels were negative.

**TABLE 1:** Patient initial laboratory results upon presentation.

Test	Result	Unit (reference range)
Hemoglobin	5.1	g/dL (13–17)
WBC	3000	μL (4000–10,000)
Platelet	1144 * 10^3^	Count/μL (150 * 10^3^ – 410 * 10^3^)
Retic #	50.3 * 10^3^	Count/μL (50 * 10^3^ – 100 * 10^3^)
Retic %	1.3	% (0.5–2.5)
Peripheral smear	Severe microcytic hypochromic anemia with dimorphic picture There is leukopenia with mild neutropenia and few reactive lymphocytes and few hypersegmented neutrophils noted Platelets are markedly increased with few large forms seen	
PT	15.4	Seconds (9.4–12.5)
APTT	29.7	Seconds (25.1–26.5)
Protein S activity	52.2	% (72–126)
Protein C activity	61.3	% (70–140)
Antithrombin III activity	68.4	% (79.4–112)
Lactate dehydrogenase	278	U/L (135–225)
Haptoglobin	44	mg/dL (30–200)
Ferritin	2.3	μg/L (48–420)
Fe% Saturation	3	% (15–45)
Iron	2	μmol/L (6–35)
TIBC	75	μmol/L (45–80)
Transferrin	3	g/L (2–3.6)
C‐reactive protein	0.7	mg/L (0–5)

Peripheral smear showed severe microcytic hypochromic anemia with moderate anisopoikylocytosis with few ovalocytes, few elliptocytes, few spherocytes, few polychromatophilic cells, and few tear‐drop cells. Moreover, there was leukopenia with mild neutropenia, few reactive lymphocytes, and few hypersegmented neutrophils. Platelets are markedly increased with few large forms seen.

ECG revealed a normal sinus rhythm pattern. CT Angiogram of the Aorta and the lower limbs was unremarkable, with no evidence of luminal irregularity or narrowing of the lower limb arterial system. A bedside transthoracic echocardiography with contrast revealed a normal left ventricular ejection fraction, without any wall motion abnormality, vegetation, or left ventricle thrombus. Furthermore, he underwent MRI brain to rule out ischemic stroke which was unremarkable for any acute brain insults.

The patient was managed conservatively with intravenous fluids, Aspirin, and prophylactic dose of enoxaparin. He was also started on morphine and pregabalin for pain management and transfused with two units of packed red blood cells. Afterward, he received 1000 mg of IV Ferric Carboxymaltose to replete his iron stores.

## 3 CONCLUSION AND RESULTS

**FIGURE 2:**
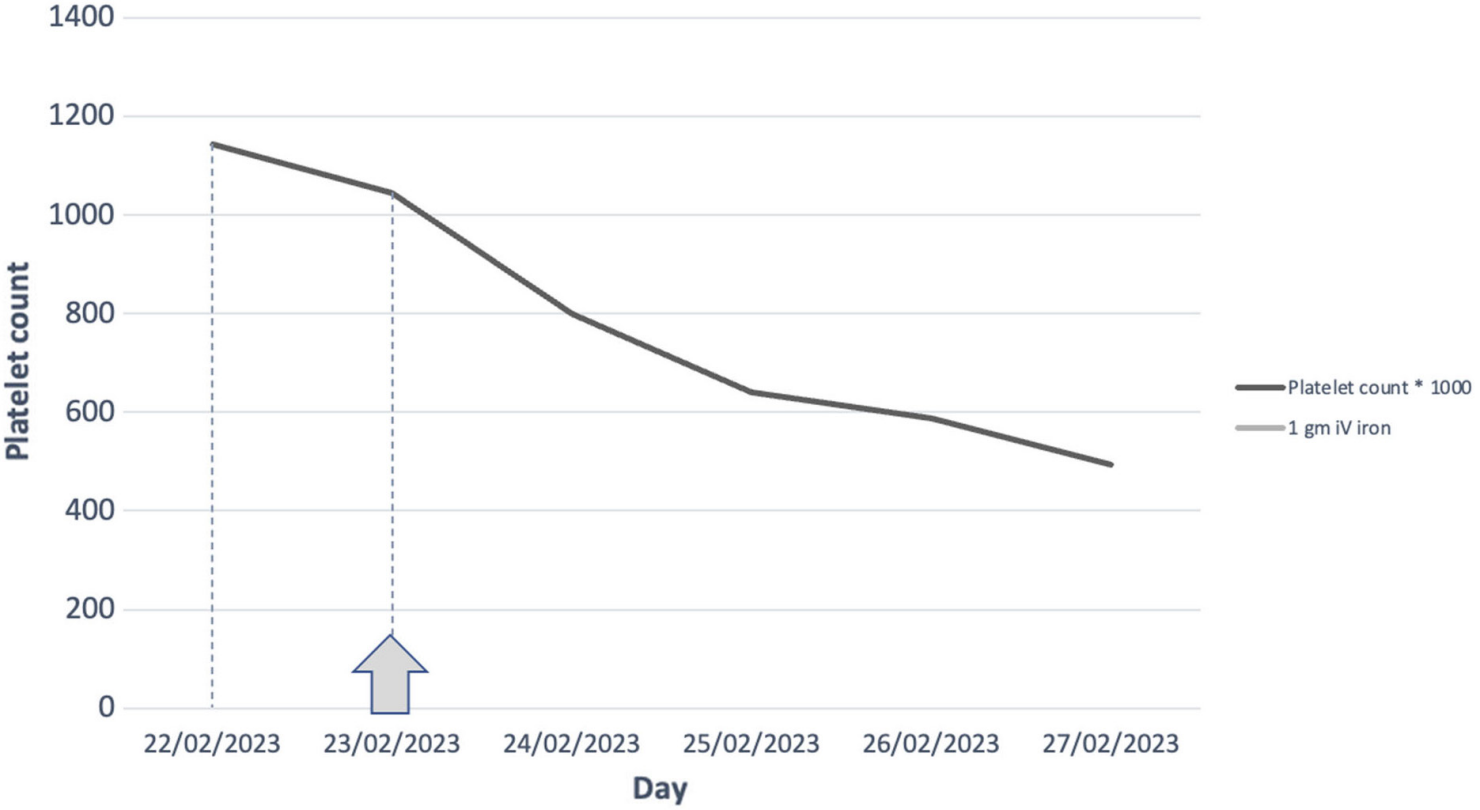
Platelet count trend during hospitalization with relation to iron replacement therapy.

He was subsequently hospitalized for observation and to investigate for any underlying source of emboli such as Infective Endocarditis or thrombophilia, but all work‐up was unremarkable. Moreover, the discoloration and pain significantly improved after 3 days of aspirin and iron replacement. Furthermore, the thrombocytosis progressively improved over the 3 days following iron replacement and IV fluids. He was discharged with a hemoglobin of 7.7 g/dL and a platelet count of 493,000 per microliter (Figure 2). His discharge medications included Aspirin 100 mg once daily and 500 mg of IV Ferric Carboxymaltose, scheduled for 1 week after the first Ferric Carboxymaltose injection given during his hospital stay.

During a follow‐up appointment after 2 months of repletion of iron stores with two doses of ferric carboxymaltose the patient was pain‐free. Complete blood count showed improvement of hemoglobin back to normal level (14.9 g/dL). Platelet count was also back to normal level (246,000/Ul). The patient was given follow‐up appointments with bariatric medicine for maintenance therapy and monitoring of hemoglobin and platelets.

## 4 DISCUSSION

Hirschmann et al. have provided a comprehensive analysis of the multiple factors that contribute to the reduced blood circulation or vascular damage underlying BTS.^3^ These factors have been categorized into three main classifications, namely diminished arterial perfusion, impaired venous outflow, and abnormal circulating blood. Notably, thrombocythemia represents one potential cause of abnormalities in the circulating blood and warrants consideration in the differential diagnosis of BTS.^3^ Considering the extensive range of potential diagnoses for BTS, the identification of BTS as a secondary manifestation of thrombocytosis was determined following the careful exclusion of alternative etiological factors for BTS in the presented patient, as illustrated in Table 2.

**TABLE 2:** Causes of blue toe syndrome and reason for exclusion in this patient.

Cause/differential diagnosis	Reason for exclusion
Decreased arterial flow Embolism Atheroembolic Thrombosis Vasoconstrictive disorders Medication‐induced vasoconstriction	Normal capillary oximetry. (SPO2 97%) Normal filling of contrast on CT Angiogram of the lower limbs arterial system with no narrowing or constriction Palpable peripheral pulses
Cardiac or aortic tumor Myxoma Cardiac vegetations Infective endocarditis Pyogenic infection	No evidence of mass on ECHO No history of fever Negative sepsis work‐up (Negative blood cultures, normal CRP, and Procalcitonin)
Antiphospholipid syndrome	Negative Lupus Screen (Negative ANA, Negative Lupus anticoagulant, Negative Anti‐cardiolipin antibodies, Negative B2 Glycoprotein antibodies)
Disseminated intravascular coagulation Thrombotic thrombocytopenic purpura Thrombophilia	Normal Coagulation Panel (INR of 1.3, APTT of 29.7 s) No thrombocytopenia No evidence of markedly decreased protein C protein S, Normal Antithrombin III activity)
Acrocyanosis	Pain present, no hypothermia or sweatiness
Syphilis	No history suggestive of Syphilis and no suspicious sexual contact
Bechet's disease	No previous history of Bechet's Disease. No mouth or genital ulcers, ocular symptoms, or rashes
Myeloproliferative disorders	Unlikely as hemoglobin and Platelet count responded rapidly to Iron therapy, follow‐up CBC showed normal hemoglobin and platelets
Polycythemia vera	Unlikely due to hematocrit of 20.2

The diagnostic process for identifying the underlying cause of thrombocytosis involves a systematic approach that encompasses sequential testing for prevalent etiological factors. According to Bleeker et al., reactive thrombocytosis is the most frequently observed form of thrombocytosis in clinical settings.^2^ Notably, iron deficiency anemia is a common trigger for reactive thrombocytosis, and therefore, an evaluation of ferritin levels and iron studies should be an integral part of the assessment for individuals with suspected reactive thrombocytosis. The diagnosis of IDIT ultimately relies on clinical judgment based on laboratory findings and the subsequent resolution of thrombocytosis following iron replacement therapy.^2^ In the presented case, the patient exhibited a positive response with a normalization of platelet count upon the treatment of their iron deficiency. Consequently, further testing for alternative causes of thrombocytosis was deemed unnecessary. Moreover, other sinister consequences of thrombocytosis such as stroke were ruled out by a brain MRI. Nevertheless, the short duration of neurological symptoms such as left‐sided weakness and numbness could represent a transient ischemic attack.

Thrombocytosis, particularly in the setting of extreme thrombocytosis (platelet count >1000 × 10^9^), poses a heightened risk of microvascular and macrovascular thrombotic events. Although reactive thrombocytosis is generally considered a self‐limiting condition with a relatively low likelihood of thrombotic complications, instances of such events have been reported. These findings underscore the associated morbidity of reactive thrombocytosis and emphasize the importance of prompt diagnosis and treatment. In the presented case, the patient exhibited extreme thrombocytosis at the time of presentation, significantly elevating the risk of thrombotic events and ultimately leading to the development of BTS.

Iron deficiency can arise from various factors, such as inadequate iron intake, compromised absorption in the gastrointestinal tract, elevated iron demands, systemic illnesses, or multifactorial.^1^ In the presented case, the history of sleeve gastrectomy, which hampers iron absorption in the gastrointestinal tract, is the most probable contributor to the iron deficiency. The pathophysiological mechanisms underlying reactive thrombocytosis in the context of iron deficiency anemia remain incompletely comprehended. Among several hematopoietic cytokines studied, only erythropoietin (EPO) exhibited a significant elevation in patients with IDIT, and this elevation in EPO levels subsequently decreased following the administration of iron replacement therapy.^2^ The elevation of EPO levels in response to iron deficiency is thought to be regulated through the modulation of the hypoxia‐inducible factor (HIF) pathway.^4^ The activation of the HIF pathway is contingent upon its unique role as a sensor for both iron and hypoxia.^4^ While it has been posited that EPO may act in synergy with thrombopoietin (TPO) to increase platelet production, it has also been suggested that the relationship between iron deficiency and reactive thrombocytosis is more complex than a sole consequence of a cross‐reactivity between EPO and TPO which is out of scope of the current discussion.^2^, ^5^ Furthermore, aside from the elevated risk of thrombosis associated with thrombocytosis, another hypothesis proposes that the reduced antioxidant defense observed in individuals with iron deficiency anemia may contribute to heightened oxidative stress, thereby fostering a propensity for platelet aggregation.^5^

The review conducted by Franchini et al. encompassed several documented cases of IDA, with or without thrombocytosis, associated with diverse thrombotic events.^5^ These events included stroke, cerebral venous thrombosis, occlusions of the central retinal artery and vein, as well as carotid artery thrombosis. Subsequent to this review, multiple reports have emerged, detailing additional cases where IDA was linked to thrombotic occurrences such as recurrent pulmonary embolism, deep vein thrombosis, aortic arch, and abdominal aortic thrombosis, as well as ischemic stroke, among others.^6^, ^7^, ^8^, ^9^, ^10^ The management of IDIT entails targeting the underlying cause of IDA, typically through iron supplementation, which results in a rapid reduction in platelet counts.

The case presented sheds light on iron deficiency anemia (IDA) as a potential etiology for blue toe syndrome (BTS). It is imperative to emphasize that the timely identification and suitable management of the underlying cause of BTS are pivotal in preventing the advancement to severe ischemic consequences. Neglecting to address BTS promptly can result in complications, such as ulceration, tissue deterioration, infection, and the potential development of gangrene, which may necessitate amputation.^3^

## AUTHOR CONTRIBUTIONS

**Khaled Aboujabal:** Conceptualization; data curation; investigation; writing – original draft. **Khaled Sadek:** Conceptualization; formal analysis; investigation; supervision; writing – original draft; writing – review and editing. **Koutaibah Obaid:** Conceptualization; formal analysis; writing – original draft; writing – review and editing. **Bisher Sawaf:** Formal analysis; investigation; visualization. **Elmukhtar Habas:** Supervision; validation. **Mhd Baraa Habib:** Data curation; methodology; supervision; writing – review and editing.

## FUNDING INFORMATION

This research did not receive any specific grant from funding agencies in the public, commercial, or not‐for‐profit sectors.

## CONFLICT OF INTEREST STATEMENT

The authors report no conflict of interest.

## CONSENT

Written informed consent was obtained from the patient for publication of this case report and any accompanying images.

## ETHICAL APPROVAL

Ethical Approval was obtained by the Medical Research Center (MRC) under ID MRC‐04‐23‐155.

## Data Availability

Data can be obtained from the corresponding author upon request.
